# Dose-related nephrotoxicity of carboplatin in children

**DOI:** 10.1038/sj.bjc.6690697

**Published:** 1999-09

**Authors:** M W English, R Skinner, A D J Pearson, L Price, R Wyllie, A W Craft

**Affiliations:** Sir James Spence Institute of Child Health, The Royal Victoria Infirmary, Newcastle upon Tyne, NE1 4LP, UK

**Keywords:** carboplatin, children, renal function, nephrotoxicity, adverse effects, chemotherapy

## Abstract

This study investigated changes and the time course of these changes in renal function in children following treatment with carboplatin, and identified risk factors for nephrotoxicity. Glomerular and proximal renal tubular function were investigated before and up to 2 years after treatment in 23 children who received carboplatin. The main findings were reduced glomerular filtration rate (GFR), and increased renal tubular loss of magnesium, manifested by a low serum magnesium (S Mg). The mean fall in GFR was 22 ml min^−1^ 1.73 m^−2^ (*P* = 0.012), and in S Mg it was 0.17 mmol l^−1^ (*P* = 0.0077). No patient had a clinically important reduction in GFR, and only one patient had symptomatic hypomagnesaemia. GFR and S Mg did not change over time after completion of treatment. Cumulative dose (CD) of carboplatin was inversely related to mean S Mg at the end of treatment (*P* = 0.031), and directly related to the fall in S Mg (*P* < 0.001). Calculated cumulative area under the plasma concentration versus time curve (AUC) of carboplatin was inversely related to S Mg after treatment (*P* = 0.004). Dose intensity (DI) of carboplatin was not shown to be related to S Mg following treatment. CD, AUC and DI of carboplatin were not related to GFR, nor change in GFR, after treatment. High CDs of carboplatin may be associated with evidence of renal damage qualitatively similar to but less severe than that caused by cisplatin. GFR and SMg should be carefully monitored when high CDs of carboplatin are used, or if carboplatin is combined with other nephrotoxic chemotherapy. © 1999 Cancer Research Campaign


*British Journal of Cancer* (1999) **81**(2), 336–341
© 1999 Cancer Research Campaign
Article no. bjoc.1999.0697

### Dose-related nephrotoxicity of carboplatin in children

**MW English, R Skinner, ADJ Pearson, L Price, R Wyllie and AW Craft**

Sir James Spence Institute of Child Health, The Royal Victoria Infirmary, Newcastle upon Tyne NE1 4LP, UK

**Summary** This study investigated changes and the time course of these changes in renal function in children following treatment with carboplatin, and identified risk factors for nephrotoxicity. Glomerular and proximal renal tubular function were investigated before and up to 2 years after treatment in 23 children who received carboplatin. The main findings were reduced glomerular filtration rate (GFR), and increased renal tubular loss of magnesium, manifested by a low serum magnesium (S Mg). The mean fall in GFR was 22 ml min$^{-1}$ 1.73 m$^{-2}$ ($P = 0.012$), and in S Mg it was 0.17 mmol l$^{-1}$ ($P = 0.0077$). No patient had a clinically important reduction in GFR, and only one patient had symptomatic hypomagnesaemia. GFR and S Mg did not change over time after completion of treatment. Cumulative dose (CD) of carboplatin was inversely related to mean S Mg at the end of treatment ($P = 0.031$), and directly related to the fall in S Mg ($P < 0.001$). Calculated cumulative area under the plasma concentration versus time curve (AUC) of carboplatin was inversely related to S Mg after treatment ($P = 0.004$). Dose intensity (DI) of carboplatin was not shown to be related to S Mg following treatment. CD, AUC and DI of carboplatin were not related to GFR, nor change in GFR, after treatment. High CDs of carboplatin may be associated with evidence of renal damage qualitatively similar to but less severe than that caused by cisplatin. GFR and SMg should be carefully monitored when high CDs of carboplatin are used, or if carboplatin is combined with other nephrotoxic chemotherapy.

© 1999 Cancer Research Campaign

**Keywords:** carboplatin; children; renal function; nephrotoxicity; adverse effects; chemotherapy

Carboplatin is a second-generation platinum compound which was developed in an attempt to overcome side-effects such as renal damage, peripheral neuropathy and ototoxicity, which were associated with its parent compound cisplatin. It is now a first-line drug for several paediatric tumours including germ cell tumours (Pinkerton et al, 1990), primitive neuroectodermal tumours and low-grade gliomas (Lashford et al, 1996), neuroblastoma (Castel et al, 1995) and malignant mesenchymal tumours (Doz and Pinkerton, 1994), and has demonstrated activity in other malignancies including Wilm's tumour (de Camargo et al, 1994; Ettinger et al, 1994).

Initial reports of the effect of carboplatin on renal function in children indicated little or no impairment in glomerular filtration following carboplatin (Castello et al, 1990; Pinkerton et al, 1990; Stevens et al, 1991; Brandt and Broadbent, 1993), even when doses of 1000 mg m$^{-2}$ course$^{-1}$ were used (Castello et al, 1990). Hypomagnesaemia has been reported after carboplatin in children (Skinner et al, 1991*a*; Ettinger et al, 1994). There has been one case report of acute renal failure following high-dose carboplatin in a child (Frenkel et al, 1995).

The purpose of this investigation was to examine renal function in a cohort of children who had completed treatment with carboplatin, to determine whether renal function changed over time after treatment, and to identify possible risk factors for nephrotoxicity.

*Received 3 December 1998*
*Revised 25 March 1999*
*Accepted 12 April 1999*

*Correspondence to:* MW English, The Department of Paediatric Haematology and Oncology, Llandough Hospital, Penlan Road, Penarth CF64 2XX, UK

### MATERIALS AND METHODS

### Patients

Twenty-three patients (ten female) were studied. All had received carboplatin but not cisplatin or ifosfamide at the Children's Cancer Unit, Newcastle upon Tyne between 1988 and November 1994. Their median age at diagnosis was 4.6 years (range 0.4–15.4 years). The carboplatin dose (mg m$^{-2}$) along with concurrent heights, weights and GFR measured by $^{51}$Cr–EDTA clearance, together with details of any treatment with nephrotoxic antibiotics or chemotherapy, were recorded. In 11 children $^{51}$Cr–EDTA clearances were performed at least once every second course of chemotherapy which enabled the cumulative area under the plasma concentration versus time curve (AUC) of carboplatin for all courses to be calculated. Carboplatin AUC was calculated according to the paediatric dosing equation (Newell et al, 1993):

AUC [mg/ml.min] = Dose [mg] / (GFR [ml/min] + (0.36 × BW [kg])) where BW equals the body weight.

Patients were treated on a variety of different protocols. Three received treatment with high-dose methotrexate, but no other nephrotoxic anticancer agents were employed. Four children received treatment with aminoglycoside antibiotics and four received amphotericin B during supportive care after chemotherapy. The case records of one child were not available, but it is known that she did not receive any nephrotoxic chemotherapy other than carboplatin. The median cumulative dose (CD) of carboplatin was 2590 mg m$^{-2}$ (range 1364–7133 mg m$^{-2}$). Patient characteristics and details of carboplatin doses administered are summarized in Table 1.

**Table 1** Characteristics of patients studied

| Patient | Age (years) | Sex | Diagnosis | Investigations | Total dose of carboplatin (mg m$^{-2}$) | Dose intensity of carboplatin (mg m$^{-2}$) week$^{-1}$) | Mean carboplatin dose each course (mg m$^{-2}$) | Other nephrotoxic treatment |
|---|---|---|---|---|---|---|---|---|
| 1 | 15.1 | M | Glioma | 4 y | 7133 | 124 | 594 | |
| 2 | 1.8 | F | Glioma | 1 m | 5239 | 135 | 524 | |
| 3 | 13.1 | F | Dysgerminoma | 1 m, 2 y | 4063 | 180 | 677 | AG |
| 4 | 0.6 | M | Astrocytoma | 6 m, 2 y | 1364 | 196 | 742 | AG |
| 5 | 5.6 | F | PNET | 1 m, 2 y | 2294 | 167 | 1148 | AG, Ampho |
| 6 | 3.8 | F | PNET | 1 m | 2324 | 171 | 1162 | |
| 7 | 4.6 | M | PNET | 1 m | 2365 | 197 | 1182 | |
| 8 | 1.4 | F | Sacrococcygeal teratoma | 1 m, 2 y | NK | NK | NK | NK |
| 9 | 0.6 | M | Neuroblastoma | 6 m, 1 y, 2 y | 1364 | 222 | 682 | AG |
| 10 | 0.8 | F | Sacrococcygeal teratoma | 1 m, 1 y, 2 y | 3967 | 214 | 661 | |
| 11 | 0.9 | F | Retinoblastoma | 1 m, 2 y | 1815 | 153 | 454 | |
| 12 | 12.1 | M | Hypothalamic teratoma | 1 m, 1 y | 3855 | 156 | 550 | Ampho, MTX |
| 13 | 12.3 | F | Hypothalamic teratoma | P, 1 m, 1 y | 2766 | 164 | 614 | Ampho, MTX |
| 14 | 12.2 | M | PNET | P, 1 m, 1 y | 2389 | 199 | 1194 | |
| 15 | 11.6 | M | Pineal teratoma | P, 1 m, 6 m, 1 y | 3560 | 106 | 508 | Ampho, MTX |
| 16 | 4.7 | M | PNET | P, 1 m, 1 y | 2114 | 154 | 1056 | |
| 17 | 1.2 | M | Low grade astrocytoma | P, 1 m, 1 y | 5485 | 141 | 499 | |
| 18 | 15.4 | M | Hypothalamic teratoma | P, 1 m, 6 m | 6006 | 282 | 1001 | |
| 19 | 1.4 | M | Teratoma | 6 m, | 2413 | 192 | 528 | |
| 20 | 1.8 | F | Low-grade astrocytoma | 6 m | 7133 | 179 | 634 | |
| 21 | 16.2 | M | Pineal dysgerminoma | P, 1 m, 6 m | 3801 | 166 | 603 | |
| 22 | 10.9 | F | Low-grade astrocytoma | P, 1 m, 6 m | 2063 | 143 | 1032 | |
| 23 | 0.4 | M | Teratoma | P, 1 m | 2222 | 171 | 555 | |
| Median | | | | | 2590 | 169 | 648 | |
| Min | | | | | 1364 | 106 | 454 | |
| Max | | | | | 7133 | 282 | 1194 | |

PNET = primitive neuroectodermal tumour. P = pretreatment, 1 m = 1 month, 6 m = 6 months, 1 y = 1 year, 2 y = 2 years, 4 y = 4 years. AG = aminoglycoside antibiotics or vancomycin; Ampho = amphotericin; MTX = high dose methotrexate; NK = not known.

### Methods

### *Investigation of renal function*

We have previously described a protocol for the detailed evaluation of glomerular, proximal and distal renal tubular function (Skinner et al, 1991*b*). Glomerular filtration rate (GFR) was measured from $^{51}$Cr-EDTA plasma clearance. A GFR < 90 ml min$^{-1}$ 1.73 m$^{-2}$ was considered to be below normal. Corresponding plasma and urine specimens are obtained and serum magnesium (S Mg), fractional excretion of magnesium (FEMg), serum ionized calcium (S Ion Ca), fractional excretion of glucose (FEgluc), and the ratios of the urine protein (Uprot:C), retinol binding protein (URBP:C), lactate dehydrogenase (ULDH:C), alanine amino peptidase (UAAP:C), alkaline phosphatase (UAKP:C) and *N*-acetyl glucosaminidase (UNAG:C) to urine creatinine were measured. Renal function was assessed 1 month, 6 months (1994 onwards), 1 year (1993 onwards) and 2 years after the completion of treatment using this protocol. Before the first course of treatment with carboplatin twenty-one children had GFR measured, but only nine had a full assessment of renal function performed.

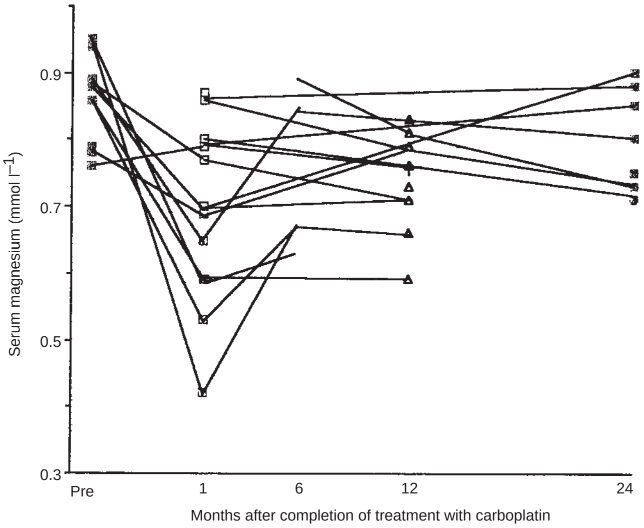

**Figure 1** Serum magnesium following treatment with carboplatin

### *Change in renal function over time*

GFR, S Mg, FEMg, S Ion Ca, FEgluc, Uprot:C, URBP:C, ULDH:C, UAAP:C, UAKP:C and UNAG:C were evaluated by paired Student's *t*-test comparing before to completion of treatment, and before to the regular time points after treatment.

*Prediction of risk factors for carboplatin nephrotoxicity*

Because there were significant changes in S Mg and GFR from before to after completion of treatment they were chosen as indices of carboplatin nephrotoxicity. As there was no significant change in the values of S Mg and GFR over time after the completion of treatment with carboplatin, the mean of all the post-treatment observations for S Mg and GFR for each patient, and the difference between the pretreatment and the mean post-treatment result for each patient were used as markers of carboplatin nephrotoxicity.

Pretreatment GFR, CD, DI, and AUC of carboplatin were chosen as potential predictors of carboplatin nephrotoxicity. DI was expressed as mg $m^{-2}$ $week^{-1}$ of carboplatin. Their importance as predictors of nephrotoxicity was evaluated by linear regression analysis.

These investigations were approved by the Joint Ethics Committee of Newcastle Health Authority and the University of Newcastle upon Tyne. Informed consent for participation was obtained from the parents and, where appropriate, the patients.

### RESULTS

### Pretreatment renal function

Three patients had slightly low and three had slightly high GFRs (79, 86 and 89, and 201, 207 and 217 ml $min^{-1}$ 1.73 $m^{-2}$). All results were confirmed as showing predicted volumes of distribution of $^{51}$Cr–EDTA within normal limits and as having good linear fits on the clearance slope of the isotope. S Mg was normal in all patients before treatment commenced.

### Change in GFR and S Mg following carboplatin

S Mg ($P$ = 0.0077) and GFR ($P$ = 0.012) both fell significantly during treatment with carboplatin. The mean reduction in S Mg was 0.17 mmol $l^{-1}$ (95% confidence interval (CI) 0.06–0.28), and the mean fall in GFR was 22 ml $min^{-1}$ 1.73 $m^{-2}$ (95% CI 5–38). Figures 1 and 2 show the changes in S Mg and GFR from before to up to 2 years after completion of treatment. GFR and S Mg did not change significantly over the 2 years following the completion of treatment. One patient had symptomatic hypomagnesaemia and suffered a fit 1 year after completion of treatment when magnesium treatment was withdrawn. It was restarted and he has been asymptomatic since then. No other patient had any clinically important symptoms which could be attributed to renal damage.

### Changes in other measures of renal function

Minor abnormalities of no clinical significance were noted for S Ion Ca, FE Mg, FE gluc, Uprot:C, URBP:C, ULDH:C, UAAP:C, UAKP:C, and UNAG:C after the completion of treatment. There was a statistically significant but clinically unimportant fall in UNAG:C between 1 month after treatment and later studies, mean fall 0.15 U $mmol^{-1}$ creatinine (95% CI 0.03–0.27).

### Prediction of risk factors for carboplatin nephrotoxicity

Increasing CD of carboplatin was inversely related to the mean S Mg after treatment ($P$ = 0.031, $r^2$ 0.212), and directly related to the reduction in S Mg over the course of treatment ($P$ < 0.001, $r^2$ 0.814) (Figure 3). DI of carboplatin was not statistically related to S Mg after treatment, or to change in S Mg from before to after treatment. However, patient 18 with both a high CD (6006 mg $m^{-2}$) and high DI

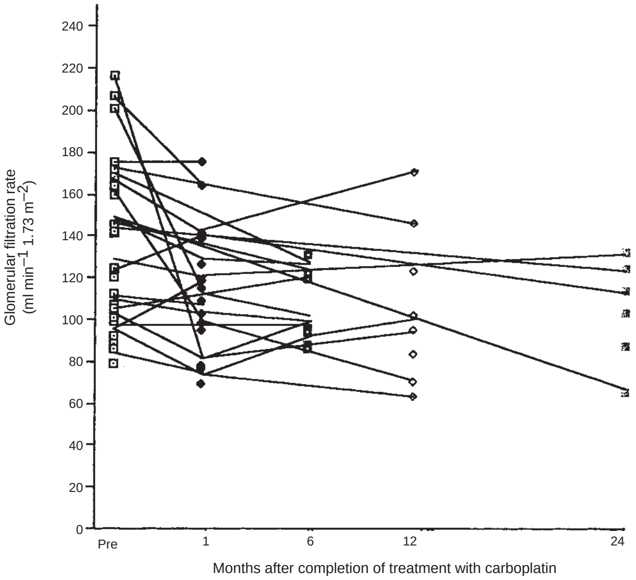

**Figure 2** Glomerular filtration rate following treatment with carboplatin

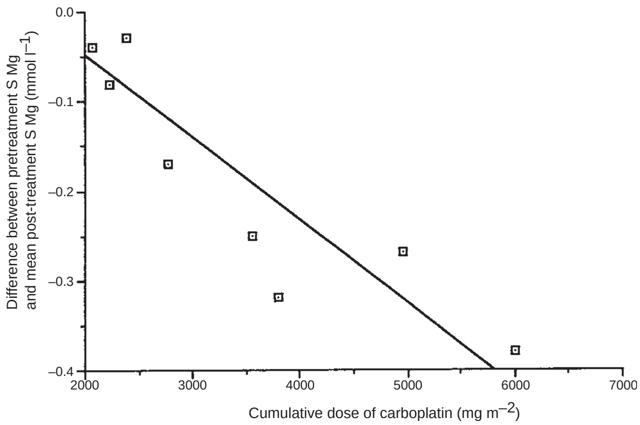

**Figure 3** Cumulative dose of carboplatin and the difference between pretreatment and mean post-treatment serum magnesium for each patient

(282 mg $m^2$ $week^{-1}$) of carboplatin had the lowest mean S Mg after treatment, whereas patients 1 and 20 had high CDs (7133 mg $m^{-2}$ each) but low DIs (124 and 179 mg $m^{-2}$ $week^{-1}$) of carboplatin and both had mean S Mg > 0.7 mmol $l^{-1}$ after treatment, so increased DI may be important with higher CDs of carboplatin. Cumulative AUC of carboplatin was inversely related to mean S Mg after completion of treatment ($P$ = 0.004, $r^2$ 0.62) (Figure 4). This result is heavily influenced by patient 18 who also had a high DI. There was insufficient data to compare cumulative AUC of carboplatin with change in S Mg over the course of treatment. CD and cumulative AUC of carboplatin were not related to GFR after the completion of treatment, nor to change in GFR from before to after treatment.

### Other risk factors for renal damage

Three patients with intracranial germ cell tumours received a protocol which combined carboplatin with intermediate dose methotrexate (1 g $m^{-2}$) 10 days later. GFR in these children was 69,

© 1999 Cancer Research Campaign

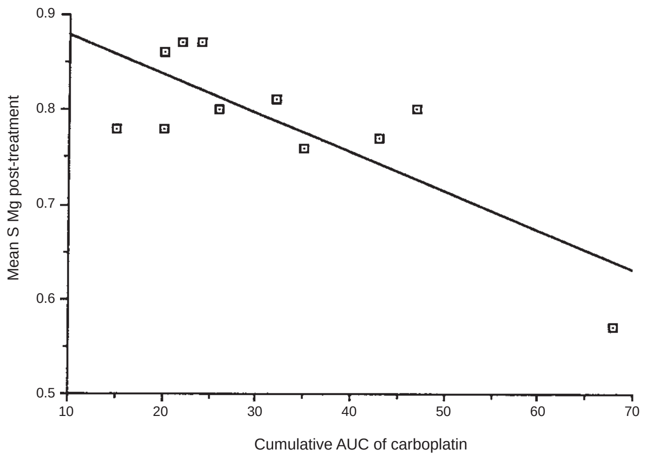

**Figure 4** Other risk factors for [illegible] Cumulative AUC of carboplatin and the m[illegible] for each patient after treatment

76 and 77 ml min$^{-1}$ 1.73 m$^{-2}$ 1 month after the completion of therapy, but compared with patients receiving similar CDs of carboplatin they did not have a disproportionate fall in GFR following treatment. Each also received amphotericin B. Only one other patient received amphotericin, and he had no recorded nephrotoxicity. The number of patients receiving aminoglycoside antibiotics was too small to permit statistical analysis, but there was no obvious association with nephrotoxicity.

### DISCUSSION

This study has shown that the predominant changes in renal function observed after treatment with carboplatin are reductions in GFR and S Mg. The reduction in GFR is seen in spite of the fact that there were five children under the age of 1 year. The change in the surface area to weight ratio over the first year of life means that when the GFR is expressed as a ratio to surface area it increases over the first year of life (there are minimal changes in the second year of life). There were insufficient younger patients to explore this further, but this phenomenon may have reduced the magnitude of the observed fall in GFR seen in this study. Qualitatively similar, but quantitatively more severe, changes are also seen after cisplatin treatment in children (Womer et al, 1985; Brock et al, 1991). Higher CD of carboplatin was significantly correlated with lower S Mg concentrations after treatment, as was higher cumulative AUC of carboplatin. A statistical relationship between DI of carboplatin and S Mg after treatment was not observed, but this investigation does not have sufficient numbers to exclude an effect at higher CDs and DIs of carboplatin.

Single doses of less than 800 mg m$^{-2}$ carboplatin have not been found to be nephrotoxic in adults (Skillen et al, 1988; Mason et al, 1991), but renal damage has been documented with higher doses. Adults with ovarian carcinoma treated with single agent carboplatin at a dose of 1000 mg m$^{-3}$ course$^{-1}$ (median of 4 courses) had a transient decrease in GFR during treatment which resolved spontaneously after therapy was completed (Hardy et al, 1990). A detailed study by Sleijfer et al also showed a 19% reduction in GFR in adults with lung cancer receiving carboplatin at doses of 400 mg m$^{-2}$ course$^{-1}$ (maximum of five courses) (Sleijfer et al, 1989). Renal toxicity became the non-haemopoietic dose-limiting toxicity when carboplatin was infused in doses of up to 2400 mg m$^{-2}$ as a single agent with haemopoietic stem cell rescue (Shea et al, 1989), and in doses of up to 1600 mg m$^{-2}$ in combination with cyclophosphamide and etoposide (Shea et al, 1992). No nephrotoxicity was seen when carboplatin to a total of 1500 mg m$^{-2}$ was given along with high-dose etoposide to adults with germ cell tumours (Nichols et al, 1992). There has also been a report of interstitial nephritis associated with renal failure in two adults with ovarian carcinoma who received intraperitoneal carboplatin (McDonald et al, 1991), but these patients had been heavily pretreated with cisplatin.

Brandt and Broadbent examined glomerular function in detail in children receiving carboplatin using $^{51}$Cr–EDTA clearance (Brandt and Broadbent, 1993). They found no significant difference between initial GFR and GFR before the final course of treatment in 26 children. However, the mean dose of carboplatin received by their patients (2438 mg m$^{-2}$) was significantly less ($P$ = 0.008, unpaired Student's $t$-test) than that given in this study (3418 mg m$^{-2}$). In addition, no investigations were carried out after the completion of treatment. Pinkerton et al reported no significant nephrotoxicity in 21 patients receiving 'JEB' (Pinkerton et al, 1990). No patient had a GFR below 90 ml min$^{-1}$ 1.73 m$^{-2}$ and although falls in GFR of > 10% were noted in three patients there was no overall significant difference between the pretreatment GFR and the last GFR recorded during treatment in their patients. They aimed to administer a carboplatin AUC of 5–6 mg ml$^{-1}$. min based on GFR measured by $^{51}$Cr–EDTA clearance. The mean dose received by their patients was around 2300 mg m$^{-2}$ with a maximum of around 3250 mg m$^{-2}$. Stevens et al showed no significant change in GFR after treatment with carboplatin to a dose of 1010 mg m$^{-2}$ in 19 patients and 2020 mg m$^{-2}$ in nine patients (Stevens et al, 1991). Again, this is a significantly lower dose of carboplatin than that received by the patients in the present study.

Frappaz et al reported one case of acute renal failure and a > 50% reduction in GFR in six out of 39 patients receiving two courses of carboplatin (800 mg m$^{-2}$/course) and etoposide for relapsed neuroblastoma (Frappaz et al, 1992). These patients had received prior nephrotoxic therapy (mean dose of cisplatin 409 mg m$^{-2}$).

Renal tubular damage resulting in hypomagnesaemia is a recognized feature of cisplatin nephropathy in adults (Bitran et al, 1982; Buckley et al, 1984; Flombaum, 1984; Hill and Russo, 1981; Salem et al, 1984) and children (Brock et al, 1991). However, few investigators have examined aspects of renal function other than glomerular filtration in children who have received carboplatin. Hypomagnesaemia has been previously reported in a subset of the patients in this study (Skinner et al, 1991*a*). Other authors have reported it as an infrequent problem during treatment in children (Ettinger et al, 1994; Tscherning et al, 1994), and more frequently in adults receiving high-dose carboplatin prior to autologous bone marrow transplant (Shea et al, 1989). Goren et al reported chronic sub-clinical renal tubular damage after carboplatin treatment (Goren et al, 1987). Until now there have been no reports of chronic hypomagnesaemia after carboplatin and the relationship between CD of carboplatin and hypomagnesaemia has not been noted.

No change in GFR or S Mg has been demonstrated over time after completion of treatment with carboplatin in the current study. Little has been published on serial changes in renal function after completion of treatment with carboplatin. Hardy et al showed a significant reduction in median GFR from 80.5 ml min$^{-1}$ before treatment to 66.0 ml min$^{-1}$ immediately after treatment in 28

patients with ovarian carcinoma (dose 1 g $m^{-2}$ $course^{-1}$, median four courses); however, there was a subsequent increase to 82 ml $min^{-1}$ at > 3 months post-treatment (Hardy et al, 1990).

Nephrotoxicity, as defined by a GFR < 90 ml $min^{-1}$ 1.73 $m^{-2}$ or a S Mg < 0.7 mmol $l^{-1}$, could not be related to any pretreatment measures of renal function in this study. Brandt and Broadbent did not report more glomerular damage after treatment in children with low GFRs prior to treatment with carboplatin (Brandt and Broadbent, 1993).

Three patients were treated on a protocol that included high-dose methotrexate. They all had low GFRs at completion of treatment, but had also required treatment with amphotericin. There was no obvious association between treatment with aminoglycoside antibiotics and nephrotoxicity. The use of amphotericin and aminoglycoside antibiotics after high-dose carboplatin has been reported to be associated with renal failure (Shea et al 1992; Marina et al, 1993). Although there have been no reports of the effect of combining carboplatin and high-dose methotrexate on renal function, the combination of carboplatin and other potentially nephrotoxic chemotherapy has been reported. High-dose carboplatin and ifosfamide prior to autologous bone marrow rescue has been associated with significant reductions in GFR, especially at higher doses of carboplatin (Broun et al, 1991; Elias et al, 1991; Wilson et al, 1992; Siegert et al, 1994), with less damage reported in children. The combination of high-dose carboplatin, melphalan, vincristine and etoposide caused severe renal toxicity when administered on the same day prior to autologous bone marrow rescue (Gordon et al, 1992) rather than spread over several days (Corbett et al, 1992).

In conclusion, treatment with carboplatin caused statistically significant reductions in GFR and S Mg in this cohort of children treated with carboplatin. However, only one patient had clinical problems requiring magnesium supplementation. Renal function was unchanged during the 2 years of observation following therapy. Hypomagnesaemia following therapy with carboplatin occurs with increasing frequency and severity after higher doses of carboplatin.

### ACKNOWLEDGEMENTS

We thank Dr M Keir and Mr Dixon Rodham for assistance with the $^{51}Cr$–EDTA clearances, Miss M Goldfinch for help with the biochemical analyses, and Dr A Skillen for the analysis of urine retinol binding protein and renal tubular enzymes. Dr Skinner was an MRC Training Fellow. This project has been supported by The Special Trustees of the Royal Victoria Infirmary, and The North of England Children's Cancer Research Fund.

### REFERENCES

Bitran JD, Desser RK, Billings AA, Kozloff MF and Shapiro CM (1982) Acute nephrotoxicity following cis-dichlorodiammine-platinum. *Cancer* **49**: 1784–1788

Brandt LJ and Broadbent V (1993) Nephrotoxicity following carboplatin use in children: is routine monitoring of renal function necessary? *Med Pediatr Oncol* **21**: 31–35

Brock PR, Koliouskas DE, Barratt TM, Yeomans E and Pritchard J (1991) Partial reversibility of cisplatin nephrotoxicity in children. *J Pediatr* **118**: 531–534

Broun ER, Nichols CR, Einhorn LH and Tricot GJ (1991) Salvage therapy with high-dose chemotherapy and autologous bone marrow support in the treatment of primary nonseminomatous mediastinal germ cell tumors. *Cancer* **68**: 1513–1515

Buckley JE, Clark VL, Meyer TJ and Pearlman NW (1984) Hypomagnesemia after cisplatin combination chemotherapy. *Arch Int Med* **144**: 2347–2348

Castel V, Badal M, Bezanilla J, Llombart A, Ruiz-Jiménez J, Sánchez de Toledo J, Melero C and Mulet M (1995) Treatment of stage III neuroblastoma with emphasis on intensive induction chemotherapy: a report from the Neuroblastoma Group of the Spanish Society of Pediatric Oncology. *Med Pediatr Oncol* **24**: 29–35

Castello MA, Clerico A, Jenkner A and Dominici C (1990) A pilot-study of high-dose carboplatin and pulsed etoposide in the treatment of childhood solid tumors. *Pediatr Hematol Oncol* **7**: 129–135

Corbett R, Pinkerton C, Pritchard J, Meller S, Lewis I, Kington J and McElwain T (1992) Pilot study of high dose vincristine, etoposide, carboplatin and high dose melphalan with autologous bone marrow rescue in advanced neuroblastoma. *Eur J Cancer* **28A**: 1324–1328

de Camargo B, Melaragno R, Silva NSE, Mendonca N, Alvares MN, Morinaka E, Marques A and Cusato MP (1994) Phase II study of carboplatin as a single drug for relapsed Wilms' tumor: experience of the Brazilian Wilms' Tumor Study Group. *Med Pediatr Oncol* **22**: 258–260

Doz F and Pinkerton R (1994) What is the place of carboplatin in paediatric oncology? *Eur J Cancer* **30A**: 194–201

Elias AD, Ayash LJ, Eder JP, Wheeler C, Deary J, Weissman L, Schryber S, Hunt M, Critchlow J, Schnipper L and et al. (1991) A phase I study of high-dose ifosfamide and escalating doses of carboplatin with autologous bone marrow support. *J Clin Oncol* **9**: 320–327

Ettinger LJ, Gaynon PS, Krailo MD, Ru N, Baum ES, Siegel SE and Hammond GD (1994) A phase II study of carboplatin in children with recurrent or progressive solid tumors. *Cancer* **73**: 1297–1301

Flombaum CD (1984) Hypomagnesemia associated with cisplatin combination chemotherapy. *Arch Int Med* **144**: 2336–2337

Frappaz D, Michon J, Hartmann O, Bouffet E, Lejars O, Rubie H, Gentet JC, Chastagner P, Sariban E, Brugiere L and et al. (1992) Etoposide and carboplatin in neuroblastoma: a French Society of Pediatric Oncology phase II study. *J Clin Oncol* **10**: 1592–1601

Frenkel J, Kool G and de Kraker J (1995) Acute renal failure in high dose carboplatin therapy. *Med Pediatr Oncol* **25**: 473–474

Gordon SJ, Pearson AD, Reid MM and Craft AW (1992) Toxicity of single-day high-dose vincristine, melphalan, etoposide and carboplatin consolidation with autologous bone marrow rescue in advanced neuroblastoma. *Eur J Cancer* **28A**: 1319–1323

Goren MP, Forastiere AA, Wright RK and et al (1987) Carboplatin (CBDCA), iproplatin (CHIP), and high dose cisplatin in hypertonic saline evaluated for tubular nephrotoxicity. *Cancer Chemother Pharmacol* **19**: 57–60

Hardy J, Tan S, Fryatt I and Wiltshaw E (1990) How nephrotoxic is carboplatin? *Br J Cancer* **61**: 644

Hill JB and Russo A (1981) Cisplatin-induced hypomagnesemic hypocalcemia [letter]. *Arch Int Med* **141**: 1100–1101

Lashford LS, Campbell RH, Gattamaneni HR, Robinson K, Walker D and Bailey C (1996) An intensive multiagent chemotherapy regimen for brain tumours occurring in very young children. *Arch Dis Child* **74**: 219–223

McDonald BR, Kirmani S, Vasquez M and Mehta RL (1991) Acute renal failure associated with the use of intraperitoneal carboplatin: a report of two cases and review of the literature. *Am J Med* **90**: 386–391

Marina NM, Rodman J, Shema SJ, Bowman LC, Douglass E, Furman W, Santana VM, Hudson M, Wilimas J, Meyer W, Madden T and Pratt C (1993) Phase I study of escalating targeted doses of carboplatin combined with ifosfamide and etoposide in children with relapsed solid tumours. *J Clin Oncol* **11**: 554–560

Mason MD, Nicholls J and Horwich A (1991) The effect of carboplatin on renal function in patients with metastatic germ cell tumours. *Br J Cancer* **63**: 630–633

Newell DR, Pearson AD, Balmanno K, Price L, Wyllie R, Keir M, Calvert AH, Lewis IJ, Pinkerton CR and Stevens MC (1993) Carboplatin pharmacokinetics in children: the development of a paediatric dosing formula. *J Clin Oncol* **11**: 2314–2323

Nichols CR, Andersen J, Lazarus HM, Fisher H, Greer J, Stadtmauer EA, Loehrer PJ and Trump DL (1992) High-dose carboplatin and etoposide with autologous bone marrow transplantation in refractory germ cell cancer: an Eastern Cooperative Oncology Group protocol. *J Clin Oncol* **10**: 558–563

Pinkerton C, Broadbent V, Horwitch A, Levitt J, McElwain T, Meller S, Mott M, Oakhill A and Pritchard J (1990) "JEB" – a carboplatin based regimen for malignant germ cell tumours in children. *Br J Cancer* **62**: 257–262

Salem P, Khalyl M, Jabboury K and Hashimi L (1984) Cis-diamminedichloroplatinum (II) by 5-day continuous infusion. A new dose schedule with minimal toxicity. *Cancer* **53**: 837–840

Shea TC, Flaherty M, Elias A, Eder JP, Antman K, Begg C, Schnipper L, Frei E3 and Henner WD (1989) A phase I clinical and pharmacokinetic study of carboplatin and autologous bone marrow support [published erratum appears in *J Clin Oncol* 1989 **7**: 1177]. *J Clin Oncol* **7**: 651–661

*© 1999 Cancer Research Campaign*

Shea TC, Storniolo AM, Mason JR, Newton B, Mullen M, Taetle R and Green MR (1992) A dose-escalation study of carboplatin/cyclophosphamide/etoposide along with autologous bone marrow or peripheral blood stem cell rescue. *Semin Oncol* **19**: 139–144

Siegert W, Beyer J, Strohscheer I, Baurmann H, Oettle H, Zingsem J, Zimmermann R, Bokemeyer C, Schmoll HJ and Huhn D (1994) High-dose treatment with carboplatin, etoposide, and ifosfamide followed by autologous stem-cell transplantation in relapsed or refractory germ cell cancer: a phase I/II study. *J Clin Oncol* **12**: 1223–1231

Skillen AW, Buamah PK, Cantwell BM, Cornell C, W HA and Harris AL (1988) Urinary protein and enzyme excretion in patients receiving chemotherapy with the cis-platinum analogs carboplatin (CBDCA, JM8) and iproplatin (CHIP, JM9). *Cancer Chemother Pharmacol* **22**: P 228–P 234

Skinner R, Pearson A, Price L, Coulthard M and Craft A (1991*a*). Renal function after carboplatin in children. *Med Pediatr Oncol* **19**: 344

Skinner R, Pearson ADJ, Coulthard MG, Skillen AW, Hodson AW, Goldfinch ME, Gibb I and Craft AW (1991*b*) Assessment of chemotherapy-related nephrotoxicity in children with cancer. *Cancer Chemother Pharmacol* **28**: 81–92

Sleijfer DT, Smit EF, Meijer S, Mulder NH and Postmus PE (1989) Acute and cumulative effects of carboplatin on renal function. *Br J Cancer* **60**: 116–120

Stevens M, Lewis I, AJ P and Pinkerton C (1991) Carboplatin and renal function in children. *Br J Cancer* **63**: 158

Tscherning C, Rubie H, Chancholе A, Claeyssens S, Robert A, Fabre J and Bouissou F (1994) Recurrent renal salt wasting in a child treated with carboplatin and etoposide. *Cancer* **73**: 1761–1763

Wilson WH, Jain V, Bryant G, Cowan KH, Carter C, Cottler FM, Goldspiel B, Steinberg SM, Longo DL and Wittes RE (1992) Phase I and II study of high-dose ifosfamide, carboplatin, and etoposide with autologous bone marrow rescue in lymphomas and solid tumors. *J Clin Oncol* **10**: 1712–1722

Womer RB, Pritchard J and Barrat TM (1985) Renal toxicity of cisplatin in children. *J Pediatr* **106**: 659–653

*© 1999 Cancer Research Campaign*
